# Significance of exosomes in hepatocellular carcinoma

**DOI:** 10.3389/fonc.2022.1056379

**Published:** 2022-11-30

**Authors:** GuoYun Wang, GaiXiang Luo, MeiJing Zhao, HuiLai Miao

**Affiliations:** ^1^ Department of Hepatobiliary Surgery, The Second Hospital of Guangdong Medical University, Zhanjiang, China; ^2^ The First Clinical Medical College of Lanzhou University, Gansu Provincial People’s Hospital, Lanzhou, China; ^3^ Key Laboratory of Liver Injury Diagnosis and Repair, Guangdong Medical University, Zhanjiang, China

**Keywords:** exosomes, hepatocellular carcinoma, microenvironment, drug resistance, development, treatments

## Abstract

Among the most prevalent cancers in the world, hepatocellular carcinoma (HCC) has a high mortality rate. The diagnosis and management of HCC are presently hindered by difficulties in early detection and suboptimal treatment outcomes. Exosomes have been shown to play an important role in hepatocarcinogenesis and can also be used for diagnosis and treatment. In this review, we discussed the research progress on exosomes in hepatocarcinogenesis development, tumor microenvironment remodeling, treatment resistance, and immunosuppression. HCC can be diagnosed and treated by understanding the pathogenesis and identifying early diagnostic markers. This review will be a significant reference for scholars with an initial understanding of the field to fully understand the role of exosomes in the organism.

## Fundamentals of exosomes

### History of exosomes

‘Exosomes’ were first discovered by Peter Wolf (1) in 1976. Using high-speed centrifugation, he extracted tiny particles of platelet origin from plasma and named them “platelet dust”, which was the first evidence of human “exosomes”. Trams et al. (2) introduced the concept of “exosomes” in 1981 by referring to vesicles of plasma membrane origin. As reported by Johnstone et al. (3), exosomes were originally thought to be a way to eliminate cellular waste to maintain cellular homeostasis. He pioneered the “waste disposal mechanism” theory by identifying exosomes as a major means of externalizing obsolete membrane proteins. According to recent studies, exosomes mediate intercellular communication and macromolecular transfer and are involved in numerous physiological and pathological processes (4–6).

### Formation of exosomes

Exosomes are extracellular vesicles (EVs) with lipid bilayers ranging in size from 30 to 100 nm (7, 8). They comprise proteins, lipids, nucleic acids (e.g., DNA, mRNA, circRNA, miRNA, and lncRNA), and bioactive metabolites. Apoptotic vesicles (1–5 µm) and microvesicles (100–1000 nm) are other examples of EVs. There are two processes that lead to the formation of exosomes: the inward budding of the plasma membrane and the inward budging of the endosome-restricted membrane depression to form intraluminal vesicles (ILVs), resulting in multivesicular bodies (MVBs) and exosomes, respectively. Exosomes can be generated by two mechanisms, namely, the endosomal sorting complex required for transport (ESCRT) and the non-ESCRT-dependent mechanism. The ESCRT depends on four protein complexes: ESCRT-0, ESCRT-I, ESCRT-II, and ESCRT-III, whereas MVBs can still be formed without ESCRT, which relies on four transmembrane proteins, including CD9, CD63, and CD82, and ceramides (9, 10). As ESCRT proteins and/or tetra-transmembrane proteins play an important role in exosome formation and MVB transport, these proteins and their auxiliary proteins (such as Alix, TSG101, HSC70, and HSP90) can also serve as exosome markers (11). CD81 and CD63 are also common exosome markers. Exosomes are produced by many types of liver cells, including hepatic parenchymal cells (e.g., hepatocytes), non-parenchymal intrahepatic immune cells (e.g., macrophages, dendritic cells, T/B cells, and natural killer cells (NK)), and several non-parenchymal hepatic stromal cells (e.g., stellate cells) (12, 13), and the size, content, and functional impacts of exosomes from different cellular sources vary significantly.

### Exosome release and binding to target cells

The smooth release of MVBs is associated with their internal cholesterol, Ca^2+^, acidic environment, and proteins (5, 14). For instance, MVBs with a high cholesterol level can fuse with the plasma membrane, while MVBs with a low cholesterol level are transported to the lysosomes for degradation (9, 15). Furthermore, elevated intracellular Ca^2+^ levels can stimulate exosome release by regulating the fusion of MVB plasma membranes. Munc13–4 is a Ca^2+^-dependent SNAP receptor crucial for Ca^2+^-dependent membrane fusion and Rab binding protein, and it can regulate exosome release (16). Rab2b, Rab5a, Rab9a, Rab27a, and Rab27b are Rab GTPases involved in exosome release, along with other GTPase families, such as Rho/Rac/cdc42. As demonstrated by Tian et al. (17), exosome release is increased in early hepatocellular carcinoma (HCC) patients in an acidic environment with high expression of miR-21 and miR-10b. As compared with non-acidic HCC-derived exosomes, acidic exosomes significantly enhanced proliferation, migration, and invasion of recipient cells, indicating a crucial role of an acidic environment in enhancing exosome release. Because early tumor tissues lack adequate perfusion, they are often hypoxic and malnourished, and the intracellular energy supply depends mostly on anaerobic enzymes, which increase lactate production and acidify the tumor microenvironment (TME) under hypoxic/hypoxic conditions. However, despite enough oxygen, tumor cells tend to produce anaerobic enzymes, a phenomenon called the “Warburg effect” (18). Moreover, hypoxia can increase cell surface receptors and ceramide expression by modulating hypoxia-inducible factors (19).

**Figure 1:**
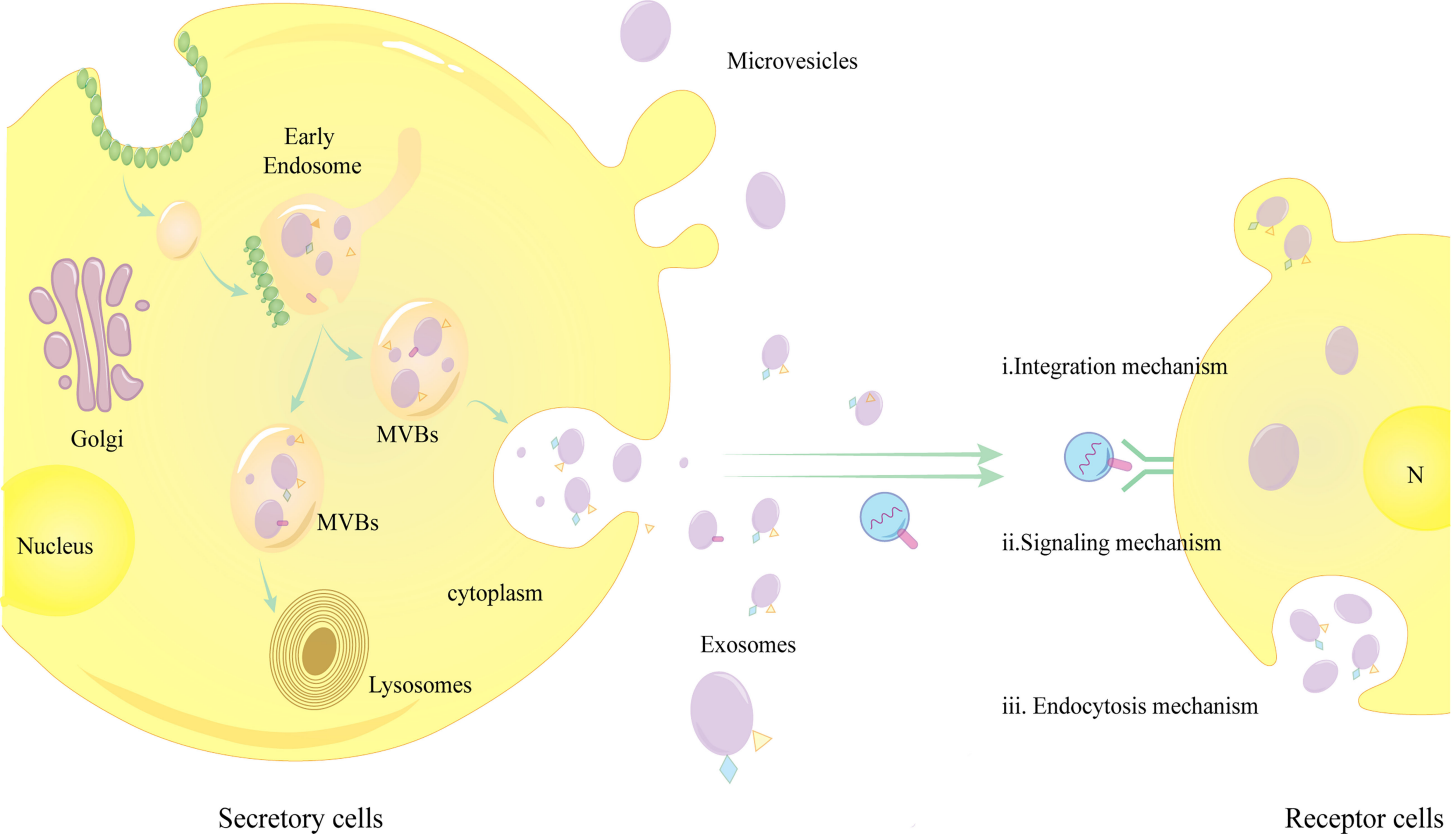
Exosome release and binding to target cells.

There are three main mechanisms through which exosomes bind to receptor cells:a,Endocytosis mechanism: Exosomes are internalized by receptor cells *via* endocytosis, which requires the cooperation of the unconventional lipid LBPA and the accessory protein Alix;b,Integration mechanism: Fusion and internalization of exosome membranes with receptor cell membranes; c,Signaling mechanism: Transmembrane proteins on exosomes act on signaling molecules on the surface of receptor cell membranes to activate intracellular signals (5, 20) **(** **Figure 1** **)**.The role of exosomes binding to the recipient cells is to regulate several physiopathological processes, such as HCC growth, invasion, metastasis, immunity, epithelial-mesenchymal transition (EMT), tumor angiogenesis and trogocytosis etc.

## Exosomes regulate the growth of HCC

“Molecular sponge mechanism” explains how overexpressed circRNAs in exosomes often act as competing endogenous RNAs (ceRNAs), suppressing miRNA expression. miRNAs negatively regulate gene expression and interfere with biological processes by blocking the translation of target mRNAs or directly destroying them, thereby contributing to cancer development. According to Lyu et al. (21), exosomal CircWHSC1 was highly expressed in HCC patients and negatively correlated with survival rate. They furtherly identified that HOXA1, a target gene of miR-142–3p, could promote HCC cell proliferation and viability. The “spongy” adsorption of miR-142–3p by CircWHSC1 increased the expression level of HOXA1 in HCC, exerting a pro-tumor effect, while CircWHSC1 silencing had an antitumor effect. Moreover, exosomal CircCdr1 overexpression as a molecular sponge inhibits miR-1270 expression, increases AFP levels (a biomarker of HCC), and promotes HCC cell proliferation and migration (22). HCC can also be protected and inhibited by exosomal circRNA in addition to the harmful effects listed above. For example, a study by Chen et al. (23) found that circ-0051443 was highly expressed in HCC tissues and plasma and that normal cells delivered circ-0051443 *via* exosomes to HCC cells, where it binds competitively to miR-331–3p, stimulating apoptosis, arresting cell division, and inhibiting HCC growth.

Sparc/osteonectin, cwcv, and kazal-like domains proteoglycan 1 (SPOCK1) is a gene encoding a seminal plasma proteoglycan. SPOCK1 is a highly conserved proteoglycan under normal physiological conditions that regulates the extracellular matrix (ECM) and is mainly expressed in the brain, cartilage, testis, heart, and vascular endothelium. Several studies show that SPOCK1 expression is upregulated in HCC tissues and cell lines, SPOCK1 promotes proliferation, colony formation, migration, invasion, and EMT of HCC cells, and high expression of SPOCK1 in the organism often correlates with a short overall survival (OS) (24, 25). By interacting with miR-940, the exosomal LncRNA THEMIS2–211 promotes SPOCK1 expression, which promotes proliferation and metastasis of HCC cells, with the opposite result after the knockdown of LncRNA THEMIS2–211 (26).

Besides nucleic acids, proteins in exosomes can also affect cancer development through several pathways. For instance, exosome-derived α-enolase (ENO1) is highly expressed in HCC cells and tissues and stimulates HCC growth and migration by upregulating integrin α6β4 expression and triggering the integrin-mediated FAK/Src-p38MAPK pathway (27). HMGB1 (High mobility group protein B1) promotes HCC progression by impairing the function of CD8+ effector T cells by triggering TIM-1+ B cell expansion, inducing TNF-α and IFN-γ factor secretion, and inhibiting CD8+ T cell proliferation (28). Through a peroxisomal mechanism, the exosomal protein LOXL4 activates the FAK/Src signaling pathway, regulates cellular matrix adhesion, and promotes tumor proliferation and migration (29). Exosomes rich in Golgi membrane protein 1 (GOLM1/GP73) induce HCC progression by activating the GSK-3β/MMPs signaling pathway in recipient cells and promoting the proliferation, migration, and invasion ability of target cells (30). In summary, all exosome-associated components are able to regulate the biological process of HCC.

## Exosomes regulate metastasis of HCC

The most common cause of clinical death from malignancy is tumor metastasis. There are numerous factors that can contribute to the spread of liver metastases, including tumor-derived factors, hormonal and signaling factors, microbial products, drugs, dietary factors, surgical procedures and their complications, and even disruption of the liver and its inter-organ communication network and inflammation.

Metastasis in HCC is largely dependent on TME formation. Once the TME develops, it allows cancer-derived cells and molecules to spread efficiently, which may cause a systemic response, such as paraneoplastic syndrome. TME can also facilitate the metastasis of circulating tumor cells (CTCs) through “pre-metastatic communication between organs” mechanisms, such as the formation of pre-metastatic ecological niches before CTCs reach their target organs (31). Mesenchymal cells, including fibroblasts, macrophages, endothelial cells, and adipocytes, play a significant role in the formation of TME (32). In TME, exosomes are mediators of intercellular communication. In cooperation with soluble molecules released by cancer and invasive cells, exosomes stimulate target cells to provide matrix components of the TME, such as exosomes transferring proteases MMP-9 and MMP-13 to the ECM. Collagen and fibrillar in the matrix are degraded by these proteases. Furthermore, exosomes release cytokines to recruit mesenchymal cells and hematopoietic cells to support the survival and proliferation of tumor cell. From existing studies, it is unclear how the pre-metastatic ecotone is regulated in the organism. Therefore, it is important to study the role of mesenchymal cells in TME to understand the pathogenesis and guide the diagnosis and treatment of HCC.

### Non-immune cells in TME and tumor migration

#### Cancer-associated fibroblasts

CAFs, commonly considered activated fibroblasts in tumor tissues, constitute a critical cellular component of the TME. They can be derived from multiple cell types, including fibroblasts, bone marrow mesenchymal stem cells, adipocytes, epithelial cells (EMT), endothelial cells (endothelial-mesenchymal transition), and stellate cells. CAFs deposit essential ECM proteins (e.g., collagen, fibronectin, and laminin) and secrete ECM degrading enzymes (e.g., matrix metalloproteinases), which induce the migration of CAFs and the degradation of ECM; thereby allowing the invasion of tumor cells. Furthermore, CAFs can mediate tumorigenesis and tumor development by secreting several cytokines. According to Fang et al. (33), highly metastatic HCC cells secrete exosomes with large amounts of miR-1247–3p. The miR-1247–3p can directly interact with β-1,4-galactosyltransferase 3 (B4GALT3) and upregulate the β1-integrin/NF-κB signaling pathway for the conversion of normal fibroblasts into CAF. As a result, the CAF can secrete pro-inflammatory cytokines IL-6 and IL-8, which promote HCC cell metastasis. In contrast, according to Yugawa et al. (34), CAF-derived exosomes inhibited the migration and invasion of HCC, with significantly lower expression of MiR-150–3p in exosomes derived from CAFs than in normal fibroblasts (NF) and transfection of CAF exosomes overexpressing MiR-150–3p into HCC cells suppressed the invasion and migration ability.

#### Endothelial cells and the HCC TME

Angiogenesis is a vital step in forming the TME, in which endothelial cells play a key role. A hypoxic/acidic environment has previously been shown to induce the release of exosomal miR-21 and miR-10b, promote angiogenesis, and promote HCC proliferation and migration (17), and in fact, several exosomal factors can affect angiogenesis. Exosomal circRNA-100338 was shown to be promoting tumor metastasis by affecting the proliferation with enhanced vascular permeability, and angiogenic mimetic (VM) formation capacity of human umbilical vein endothelial cells (HUVEC) (35). Exosomes released from cancerous hepatocyte-like CD90+ HCC cells transfected with lncRNA H19 can produce and secrete VEGF (vascular endothelial growth factor) and its receptor VEGF-R1 in HUVEC, then increase the ability of HUVEC cells to align into tubular structures *in vitro*, and promote heterotypic adhesion of endothelial cells to CSC-like hepatocytes (36). Li et al. (37) found that the exosomal Long non-coding RNA small nucleolar RNA host gene 16 (SNHG16) increased GALNT1 expression *via* sponge miR-4500 to promote angiogenesis. The proliferative, migratory, and angiogenic abilities of HUVECs were enhanced after exposure to exosomes derived from HCC cells by transmitting SNHG16.SNHG16/miR-4500/GALNT1 axis *in vitro* and *in vitro* and *in vivo* mediated angiogenesis and tumor growth. Additionally, exosomal miR-200b-3p (38), and CLEC3B (39) promote angiogenesis.

#### Adipocytes and the HCC TME

There are three types of adipocytes: white, brown, and beige. The brown and beige adipose are generally used to regulate body temperature and energy. On the other hand, white adipose fat is an endocrine organ that controls human physiopathology, such as the mature adipocyte-derived exosome circ-BD. The circ-BD decreases miRNA-34a and USP7/Cyclin A2 pathway expression, both of which induce the growth of HCC cells (40, 41). Pathological factors promote the conversion of adipocytes to cancer-associated adipocytes (CAAS), which stimulate TME to create a hypoxic environment, fibrosis, abnormal ECM remodeling, metabolic changes, and increased inflammation to promote cancer progression (41). Upon contact with adjacent adipocytes, exosomes released from HepG2 cells actively internalize into adipocytes, which then stimulate inflammatory cytokine secretion, activation of Nf-kB and other kinases, and adipocyte signaling to promote HCC tumor growth and progression (42).

#### Chemokines and the HCC TME

Chemokines are small molecule proteins with chemotactic properties that can induce targeted migration in a wide range of cell types. They are structurally similar, and most of them have four conserved cysteines, which can be divided into four subfamilies, C, CC, CXC, and CX3C4, based on the relative positions of the first two cysteines near the N terminus (43). Chemokines play a crucial role in TME. CXCL8, a chemokine in the CXC family, can stimulate the growth of HCC cells directly, mediate angiogenesis in the microenvironment, and recruit neutrophils to secrete matrix metalloproteinase 9 (MMP-9), thereby inducing the infiltration of cancer cells (44). A study by Li et al. (45) demonstrated that exosomes with high levels of CXCR4 secreted by HCC Hca-F cells promoted migration, invasion, and lymphatic vessel formation in Hca-P cells. Mechanistically, CXCR4 transfer *via* exosomes to LECs (lymphatic vessel endothelial cells) promotes lymphatic vessel formation by binding to SDF-1α in the cells, which promotes MMP-9, MMP-2, and growth factor C (VEGF-C) secretion from the endothelium. Additionally, Sun et al. (46) found that bone marrow endothelial cells upregulated CX3CL1/CX3CR1 expression in HCC spinal metastases by secreting soluble CX3CL1, which was involved in Src/protein tyrosine kinase 2 (PTK2) activation of Hep3B and MHCC97H cells migration and invasion into the spine. The invasive and migratory capacities of CX3CL1 were inhibited after its expression level was reduced.

### Immune components in TME and tumor metastasis

The immune microenvironment plays an indispensable role in the tumor progression and immune response, and the deficiency of immune function is a critical factor in tumor metastasis. The immune-related cells in the TME are primarily macrophages, natural killer cells (NK), CD4+ T lymphocytes, CD8+ T lymphocytes, and DCs (47).

#### Macrophages

Tumor immunity is mediated by macrophages, which can be polarized into M1 and M2 types. M1-polarized macrophages are often activated by factors like interferon-γ (TAT1 signaling pathway) and mostly play a role in immune surveillance, while M2 macrophages are activated by Th2 cytokines, including IL-10, IL-13, and IL-4 (STAT3 signaling pathway) and can downregulate immune responses. The M2 polarized phenotype is highly expressed in the TME (48, 49). It has been shown that macrophages are able to take up the lncRNA TUC339 in HCC-derived exosomes, leading to decreased secretion of pro-inflammatory cytokines and increased secretion of inflammatory cytokines and triggering phenotypic changes in macrophages. Moreover, these phenotypically transformed macrophages can inhibit immune-mediated tumor cell death and promote tumor immune escape, thereby inducing rapid tumor growth progression (50). Liu et al. (51) found that macrophage-derived exosomes could deliver miR-92a-2–5p to HCC cells to change PHLPP/p-AKT/β-catenin signaling and enhance the invasive capacity of HCC. Alternatively, tumor-associated macrophages overexpressing miR-99b were transformed into an antitumor phenotype (M1) with increased immunosurveillance capacity, phagocytosis, and antigen presentation.

Endoplasmic reticulum (ER) stress is a common feature of tumor cells, and studies have found that ER stress can modulate tumor immunity by regulating macrophage function in the TME (52); however, the mechanism remains unclear. Liu et al. (53) identified ER stress as a possible mechanism for releasing miR-23a-3p-rich exosomes in HCC cells. By suppressing PTEN-AKT pathway expression in macrophages, these exosomes increase programmed death ligand-1 (PD-L1) levels. The upregulated PD-L1 will bind to T-cell programmed death-1 (PD-1), which in turn inhibits T-cell proliferation, resulting in T-cell dysfunction and tumor cell immune escape. Furthermore, ER-stressed HCC cells can promote cytokine expression through exosome-mediated activation of the JAK2/STAT3 pathway in macrophages, which leads to macrophage immunosuppression and tumor progression (54). Another study found that melatonin inhibits the synthesis of pro-inflammatory cytokines and reduces inflammation which is important to promote tumor invasion in the TME and that exosomes derived from melatonin-treated HCC cells downregulate PD-L1 and cytokine expression on macrophages through the STAT3 pathway, thus attenuating the immunosuppressive state of macrophages (55).

#### Natural killer cells

NK cells distinguish “self” from “non-self” antigens through surface stimulatory and inhibitory receptors that transmit activating/inhibiting signals, respectively, and the balance between these signals determines NK cell activation. NK cells can become dysfunctional through various mechanisms in order to avoid detection by tumor cells. For example, the human NKG2D ligand MICA*008 in tumor cell-derived exosomes promotes a significant downregulation of NKG2D expression on the surface of NK cells, thereby inhibiting NK cell cytotoxicity (56). A high expression of TIM-3, one of the central inhibitory receptors for NK cells, reduces the ability of NK cells to mediate antitumor immunity. The mechanism is that HCC exosomes with high circUHRF1 expression are delivered to NK cells and upregulate TIM-3 expression by sponging miR-449c-5p, which causes NK cell failure. These studies suggest blocking TIM-3 could be a novel strategy for improving NK function in cancer patients (57).

#### Dendritic cells

DCs are derived from hematopoietic precursors in the bone marrow. During their migration to nearby lymph nodes, they capture the antigen and will differentiate and mature. They will present the antigen and activate T cells in the tumor and stimulate them to mount an antitumor immune response. In order to reduce Th1 cell initiation and induce Treg differentiation, tumor exosomes upregulate suppressor molecules, downregulate immune response components, block DC migration to lymph nodes to activate T cells, reduce T cell immune responses, and promote immune evasion (58). DCs are also targets for cancer-targeted immunotherapy, and various factors can alter the immune status of tumors, thereby affecting tumor progression. CD103/CD141 dendritic cells expressing CCR7 were demonstrated to play a key role in T cell immune initiation and the transport of antigens to and from melanoma tumors in the study by et al. (59). Furthermore, modulating dendritic function is one of the most common cancer therapies as will be discussed in more detail in the following sections.

#### T lymphocytes

One of the causes of tumor immune escape is the deficiency of CD4+ and CD8+ T cells in the body. PD-L1 is one of the two ligands of PD-1 and is expressed on the surface of cancer cells, macrophages, DCs, and monocytes. PD-L1 on cancer cells targets PD-1 on T cells and causing T cell suppression and preventing further immune exclusion. Upregulation of PD-L1 is an important mechanism related to tumor immune escape. According to Chen et al. (60), GOLM1 increased PD-L1 protein levels by deubiquitinating PD-L1 through COP9 signalosome 5, and promoted PD-L1 sorting into exosomes by inhibiting Rab27b. The translocation of exosomal PD-L1 to macrophages exacerbated the suppression of CD8+ T cells in HCC by increasing intracellular PD-L1. Infiltrative tumor-associated macrophage (TAM) inhibitors, along with anti-PD-L1 therapy, can eliminate PD-L1+ TAM infiltration and neutralize CD8+ T-cell suppression in HCC. According to Yin et al. (61), the transcription factor Sal-like protein 4 (SALL4) regulates miR-146a-5p expression in HCC exosomes, and the exosomal miR-146a-5p caused M2 polarization and dysfunction in macrophages. Wang et al. (62) stated that the high expression of 14–3–3ζ (14–3–3 protein zeta) in HCC cells was delivered to TIL (tumor-infiltrating T lymphocytes) *via* exosomes and decreased T cell functions including suppression of proliferation, activation, and differentiation and could also cause T cell depletion. Fan et al. showed that HCC cells release exosomes containing PCED1B-AS1, which enhance PD-Ls expression and function by sponging hsa-miR-194–5p, whereas suppressing recipient T cells and macrophages; thereby inducing immunosuppression in HCC (63).

### Exosome involvement in EMT in HCC

EMT is the process by which epithelial cells transform into a nonpolar, intercellularly connected state and obtain a mesenchymal phenotype with enhanced migratory and invasive capabilities. The activation of EMT is a crucial step in the development of tumor metastasis. Chen et al. (64) found that the exosome Rab27a, produced by the highly metastatic human HCC cell MHCC97H, is a significant factor in reducing pulmonary and intrahepatic metastasis of HCC. Rab27a is a small GTPase that mediates exosome secretion by regulating the fusion of the plasma membrane with MVV. Rab27a knockdown prevents exosome secretion from MHCC97Hs and enhances the expression of MHCC97H mesenchymal markers (such as N-calmodulin, α-SMA, and waveform protein), reduces the expression of epithelial marker E-calmodulin, promotes EMT in HCC cells *via* MAPK/ERK signaling pathway, and induces migration, chemotaxis, and invasion of progenitor cells. Qu et al. (65) demonstrated that HCC cell-derived exosomes could decrease intracellular E-cadherin expression and increase vimentin expression by influencing the TGF-β/Smad signaling pathway, thereby inducing EMT development and tumor migration. GADD45A is a nuclear protein that interacts with CDKN1A (cyclin-dependent kinase inhibitor 1A) and PCNA (proliferating cell nuclear antigen) and is a target gene of miR-374a-5p. GADD45A contributes to genome stability, DNA repair and inhibiting cell growth.

β-catenin, a central component of the Wnt signaling pathway and transcriptional co-activator, is abnormally activated in many cancers, including HCC, and possesses a dual effect in controlling tumorigenesis and EMT. It interacts with calmodulin E (E-cadherin) and promotes the formation of adherens junctions when localized on the plasma membrane. When the Wnt signaling pathway is activated, β-catenin translocates from the cytoplasm to the nucleus and induces EMT directly or indirectly by regulating target gene expression. Han et al. (66) found that the silencing of Vps4A or CHMP4B reduced the plasma membrane localization and exosome sorting of β-catenin and promoted EMT in HCC. Meanwhile, Vps4A overexpression impaired the β-catenin signaling pathway and inhibited EMT and HCC cell motility. Furthermore, Vps4A expression was significantly correlated with the expression of numerous EMT markers in HCC tissues, and exosomal β-catenin levels were significantly lower in patients with metastatic HCC than in controls.

### TME and tumor metabolism

Adequate oxygen and nutrients (such as glucose and glutamine) are essential for tumor cell growth and metastasis. As an example of sugar metabolism, it has been mentioned that tumor cells prefer glycolysis to produce energy even under conditions of sufficient oxygen, a change in metabolism known as the Warburg effect. It allows rapid energy production and promotes the production of lipids, amino acids, and nucleotides by other metabolic pathways that promote the growth of cancer cells. In contrast, tumor cells under hypoxic conditions will metabolically reprogram the tumor microenvironment and thus promote tumor progression. For example, tumor cells produce hypoxia-inducible factor (HIF), which is an important mediator of tumor metabolism and can regulate the expression of target genes such as VEGF, glycolytic enzymes, erythropoietin, iron metabolism-related genes, glucose transporter-1(GLUT1), heme oxygenase HO2-1, inducible nitric oxide synthase, etc. (67–69). According to Morrissey et al. (70), tumor-derived exosomes can influence glycolysis *via* the NF-kB pathway enabling metabolic reprogramming in the tumor microenvironment, resulting in increased glucose uptake, and increased lactate formation. A study by Xu et al. (71) found that tumor-associated macrophage-derived exosomes could promote glucose metabolism, cell proliferation, and the malignancy of HCC by transferring lncMMPA to tumor cells, activating the glycolytic pathway, enhancing macrophage formation of M2 polarization, and increasing the malignancy of HCC. Furthermore, Yan et al. (72) investigated that miR-105 could be induced by oncoprotein MYC in cancer cells and activate MYC signaling in CAF to promote metabolic reprogramming through the following mechanisms: a, suppressing ecotropic energy consumption while supplying cancer cells; b, energy-rich metabolites(such as acetate, glutamate) from ecotropic cells promote anabolic cancer cells; c, neighboring ecotropic cells convert metabolic by-products produced by cancer into non-toxic.

### TME and tumor epigenetics

Metabolic, epigenetic, and autophagic processes are not independent in TME but complementary and interdependent (73). Metabolites can influence the transcriptional program by affecting epigenetic modifications of chromatin, of which the most typical glucose metabolite, acetyl coenzyme A, is an important product of glucose metabolism. Furthermore, acetyl coenzyme A is also a substrate for the acetylation of proteins, including histones, which serve as markers for the initiation of gene expression. The accumulation of acetyl coenzyme A increases histone acetylation and promotes the expression of growth-related genes, which promotes cell proliferation (74). EVs regulate epigenetic processes, including DNA methylation and miRNA or lncRNA regulation, in addition to the histone modifications described above. The resulting epigenetic modifications result in changes in the expression of tumor-promoting and tumor-suppressing genes (75). For example, N6-methyladenosine (m6A) methylation is one of the most common RNA modifications and is widely involved in regulating physiological and pathological processes. m6A methylation is regulated through shearing, exporting, translating, and processing to regulate transcription and protein expression, mediated adaptations to hypoxia, metabolic dysregulation, and immune cell phenotypic shifts synergistically promote the formation of immunosuppressive TME and support tumor proliferation and metastasis (76). In the study by Yang et al. (77), the 6-methylase METTL3 enhances HIF-1α expression and maintains high levels of glycolysis, thereby promoting malignant proliferation in HCC. A study by Zhao et al. (78) found that miR-144/miR-451a clusters target and reduce the secretion of hepatocyte growth factor (HGF) and macrophage migration inhibitory factor (MIF) in HCC cells through paracrine signaling, resulting in the impairment of macrophage M2 phenotype and the promotion of M1 polarization, which facilitates phagocytosis and the activation of cytotoxic T lymphocytes for antitumor activity. In a feedback loop formed by miR-144/miR-451a clusters and EZH2, the catalytic subunit of the polycomb repressor complex (PRC2), miR-144 targets EZH2 and PRC2 through histone H3K27 methylation of the promoter for miRNA gene epigenetic suppression; simultaneously, TAM remodels and promotes the progression of HCC.

### TME and autophagy

Autophagy is a physiological cellular process for the degradation and elimination of misfolded proteins and damaged organelles that function as an adaptation to starvation, development, cell death, and tumor suppression. Autophagy begins with the formation of autophagosomes and ends with fusion with lysosomes. The autophagic machinery is essential for protecting cells from damaged proteins, organelles, and microorganisms, maintaining cellular metabolism, energy homeostasis, and cell survival. In cancer biology, the regulation of autophagy plays a dual role in tumor promotion and suppression, with autophagy inhibiting tumorigenesis by suppressing cancer cell survival and inducing cell death early in tumorigenesis. On the other hand, autophagy adapts cancer cells to metabolic stress and promotes their growth, survival, invasion, and metastasis (79–81). For instance, pancreatic ductal adenocarcinomas in a nutrient-deprived environment can induce autophagy in pancreatic stellate cells, causing them to secrete alanine. This substance can serve as an alternative carbon source and fuel for entering the tricarboxylic acid cycle in pancreatic cancer cells to participate in energy and substance synthesis and thereby promote cell proliferation (82). According to the study by Fan et al. (83) the authors induced Autophagy in HCC cells through starvation or nutritional deficiency. Autophagy activated the Wnt/β-linked protein signaling pathway, promoting the expression of monocarboxylate transporter protein 1 (MCT1), whose main function is to transport pyruvate. Also, Autophagy promotes tumor invasion and metastasis, and this process can be reversed by the autophagy inhibitors 3-methyladenine (3-MA) and β-linked protein knockdown.

Anatomically, human hepatocellular carcinoma tumor tissue can be divided into cancer nests, invasion margins, and peritumor stromal areas. According to Chen et al. (84), unlike the conventional predicted results, cancer cells with significantly increased autophagy levels were mainly enriched in the invasion margin region of HCC rather than the cancer nests, which increased the preferential marginal region activation and promoted disease progression. Meanwhile, tumors upregulated autophagy levels in cancer cells through activating surrounding monocytes in concert with monocyte-derived TNF and IL1B, and monocyte-induced autophagy promoted EMT of cancer cells but also promoted tumor metastasis by activating NFKB-SNAI1 signaling pathway. Fluid shear stress (FSS), caused by plasma leakage from abnormal permeable tumor vessels and inadequate lymphatic drainage, has been identified as a pathological cause of cancer metastasis. In Su’s study (85), exposure to FSS at 1.4 dynes/cm2 promoted autophagosome formation and significantly increased the expression of autophagy-related markers beclin1 and ATG7 in HepG2 and QGY-7703 cells. Once autophagy was blocked by 3-MA, the occurrence of FSS-induced EMT and cell migration was significantly inhibited. Several studies have shown that autophagy can be controlled to improve tumor treatment outcomes. As an example, miR-541 may enhance the response of HCC to sorafenib treatment by inhibiting autophagy, as reported by Xu et al. (86).

## Exosome-mediated HCC drug resistance/tumor resistance

The term tumor resistance refers to tumors that become resistant to treatment. In many ways, resistance can be transferred from one cell to another, so it seriously affects the effectiveness of tumor therapy and is a great challenge for current tumor treatment (87).

### Chemotherapeutic drug resistance

Drug therapy for cancer is a mainstream treatment method and a therapeutic modality with a wide range of potential. Current chemotherapeutic drug research is focused on discovering the mechanisms of tumor resistance and reversing resistance. Fu et al. (88) found that drug resistance in HCC was related to exosomal microRNA-32–5p in multidrug-resistant cell lines (Bel/5-FU) and sensitive cell lines (Bel7402), HCC and paraneoplastic liver tissues. By targeting PTEN, miR-32–5p mediates multidrug resistance to activate PI3K/Akt pathway, such as 5-fluorouracil, oxaliplatin, and gemcitabine. In contrast to the above, Semaan et al. (89) showed that microRNA-214 enhances chemotherapeutic drug sensitivity in HCC, thereby improving therapeutic efficacy. Combination therapy of human cerebral endothelial cell (hCEC)-derived exosomes highly expressing microRNA-214, hCEC-Exo-214, with the anticancer agents, such as oxaliplatin or sorafenib, significantly decreased cancer cell invasion and viability in HepG2 and Hep3B cells. This therapy was much more effective than monotherapy with either agent. It may cause a decrease in P-glycoprotein (P-gp) and splicing factor 3B subunit 3 (SF3B3), which mediate drug resistance and cancer cell proliferation, respectively.

### Targeted drug resistance

As shown by Wang et al. (90), MiR-744 was downregulated in HCC tissue cell lines and exosomes derived from patients’ serum and HepG2 cells. In contrast, the proliferation of HepG2 cells when treated with miR-744-rich exosomes was significantly inhibited, and sorafenib resistance was reduced. Sorafenib chemoresistance may be regulated by miR-744 and exosomal miR-744 by targeting PAX2. Cancer stem cells (CSC) play a significant role in determining drug sensitivity. Huang et al. (91) suggested that RAB27A mediates exosome release from CSC to maintain a stem cell-like phenotype and regorafenib insensitivity. Exosomes secreted from CSC upregulate the expression of the transcription factor Nanog in non-CSC progeny and confer regorafenib resistance. Nanog depletion sensitizes non-CSCs to regorafenib in the presence of CSC exosomes. In addition, PD1 is used as a first-line HCC chemotherapeutic agent, and cancer cell-derived exosomes circUHRF1 to induce natural killer cell depletion, facilitating resistance to anti-PD1 therapy (57).

Exosomes may also play a role in tumor resistance since drugs can stimulate exosome production in tumors and promote drug efflux through exosomes (92), raising the possibility of new resistance mechanisms. Furthermore, drug resistance may also be associated with cellular autophagy (93, 94). As shown by Liang et al. (95), the cellular stress transcription factor FOXO3a plays a role in regulating cell proliferation, apoptosis, anti-stress and metabolic pathways and that FOXO3a-dependent activation of autophagy-related gene transcription and autophagic flux is a key mechanism mediating hypoxia-induced sorafenib resistance in HCC cells. Knockdown of FOXO3a inhibited sorafenib in hypoxia-induced autophagy and significantly enhanced the efficacy of sorafenib. In spite of this, such studies are not well reported and need to be further investigated.

## Exosomes and cancer treatment strategies

### Exosomes and drugs, gene vectors

Exosomes can be genetically modified to produce monoclonal antibodies on their surfaces, such as synthetic polyvalent antibodies (SMART-Exos) that can target both tumor-associated human epidermal growth factor receptors (EGFRs) and CD3 receptors on T cells. It exhibits efficient and highly specific antitumor activity by redirecting and activating T cells to attack cancer cells expressed by EGFR (96). Exosomes can be modified to carry drugs for the treatment of tumors. Using electroporation, Liang et al. (97) loaded the anti-cancer drug Norcantharidin (NCTD) into bone mesenchymal stem cell-derived exosomes (BMSC-Exos). The results showed that the BMSC-Exos-NCTD delivery system was efficiently taken up by cells and induced cell cycle arrest, reduced HCC cell proliferation, and increased apoptosis. Compared with NCTD treatment alone, BMSC-Exos-NCTD showed more significant antitumor effects. Surprisingly, BMSC-Exos-NCTD did not cause any toxicity to the body but also repaired the damaged liver tissue in liver sections. Studies have shown that miR-122 has the essential function of promoting chemosensitivity in HCC cells. Lou et al. (98) showed that transfecting adipose tissue-derived mesenchymal stem cells (AMSC) with miR-122 expression plasmids and acting them on HCC cells. Exosomes derived from ASMCs mediated miR-122 communication between AMSC and HCC cells, altered target gene expression in HCC cells. They improved the sensitivity of HCC to anticancer drugs such as 5-FU or sorafenib. Moreover, intra-tumor injection of 122-Exo significantly improved the antitumor efficacy of sorafenib against HCC in mice. They demonstrated a novel strategy to increase HCC chemosensitivity through an exosome-mediated transfer of therapeutic miR-122 from AMSCs. Additionally, Fu et al. (99) reported that genetic circuits could reprogram the host liver, resulting in siRNA synthesis and self-assembly of secretory exosomes being directed. This siRNA targeting specific tissues is effective in silencing target genes for cancer treatment, demonstrating a new trend in RNA interference therapy.

### Regulation of exosome release or uptake

Several drugs have been studied and proven to inhibit tumor progression by modulating exosome uptake and release, such as lisdexamfetamine which is approved for clinical use in the treatment of metastatic melanoma (100). A study by Ortiz et al. (101) explains this phenomenon that type I interferon (IFN) induces the expression of cholesterol 25-hydroxylase (CH25H) and that CH25H-catalyzed production of 25-hydroxycholesterol inhibits lipid membrane fusion and thus TEV uptake. Melanoma TEV promotes TEV uptake by target cells and establishes a pre-metastatic ecological microenvironment through downregulation of type I interferon (IFN) receptor and IFN-induced cholesterol 25-hydroxylase (CH25H) expression. In contrast, the anti-hypertensive drug reserpine can inhibit TEV uptake, repair IFNAR1 and CH25H and disrupt TEV-induced pre-metastatic ecological niche and formation of melanoma lung metastases by inhibiting TEV uptake. Moreover, in a study by Lu et al. (102), the authors found that in addition to the functions mentioned above, reserpine inhibited the sustained growth of angiopoietin 2 (ANGPT-2) in endothelial cells of a mouse colon cancer model, effectively inhibiting angiogenesis and colon cancer progression. At the same time, rifampin also improved the therapeutic effects of radiotherapy and chemotherapy. Thus, alone or combined with drugs targeting VEGF, Reserpine can inhibit intratumoral angiogenesis or primary solid tumor growth. In these articles, the authors also explain and add to this theory and the mechanisms involved (103, 104).

Other drugs have also been shown to modulate exosome release and uptake. According to IM et al. (105), the antimicrobial drug sulfisoxazole (SFX) inhibits multivesicular endosome biogenesis and secretion through ESCRT-dependent mechanisms. In a mouse model of breast cancer xenografts, SFX reduced the expression of proteins involved in the synthesis and secretion of small extracellular vesicles(sEVs) by modulating the expression of endothelin receptor A (ETA). Furthermore, ETA caused the co-localization of multivesicular endosomes with lysosomes, inhibiting the production of sEVs and the development of breast cancer. In a study by Zhao et al. (106), a novel anticancer mechanism of apatinib inhibition of tumor exosome release was revealed. Namely, apatinib regulated the lysosomal marker LAMP2 to promote MVB degradation in colorectal cancer cells (CRC) and can inhibit Rab11 expression from interfering with MVB transport. Moreover, apatinib inhibited MVB membrane fusion by decreasing Synapse-associated protein 23 (SNAP23) and Vesicle-associated membrane protein 2 (VAMP2) expression. Furthermore, Datta et al. (107) tested 4580 known pharmacologically active compounds and FDA-approved drugs using a rapid, high-volume robotic screening method. There were ultimately 22 drugs discovered, including antibiotics, antifungals, and anti-inflammatory agents, that can modulate biological activities related to exosome biogenesis and/or secretion. For example, the lead compounds tipifarnib, neticonazole, climbazole, ketoconazole, and triademenol were validated as potent inhibitors; and sitafloxacin, forskolin, SB218795, fenoterol, nitrefazole, and pentetrazol were validated as activators of exosome biogenesis and/or secretion in PC cells. Unfortunately, similar drugs have not been found in HCC.

### Exosomes and cancer vaccines

Cancer vaccines are designed to stimulate the body to produce an immune response against specific cancer by using tumor cell-associated antigens. Unlike the preventive role of conventional vaccines, cancer vaccines focus on treating cancer. Exosomes’ high histocompatibility, optimal size, and circulating stability make them ideal carriers for cancer vaccines. Meanwhile, exosomal vaccines have the advantages of structural stability, high safety, easy preservation, suitability for mass production, and can improve the specificity of the immune response. DCs are natural, good antigen-presenting cells that link innate and adaptive immunity in the human body. Therefore, dendritic cell-derived exosomes are the most common cancer vaccine composition (108). Exosomes isolated from DCs are antigen-presenting cells that play a key role in the adaptive immune system. They can carry MHC class I- or MHC II-peptide complexes, heat shock proteins (HSP), and CD86, which would facilitate the recognition and activation of CD4 or CD8 T cells (108, 109). In addition to dendritic cell vaccines, cancer vaccines include cellular vaccines, DNA vaccines, mRNA vaccines, peptide vaccines, nanovaccines, etc.

The nature of immune adjuvants determines the type, scope, quality, and intensity of the antitumor immune response, as adjuvants can enhance the immunogenicity of tumor antigens, promote the presentation of tumor antigens, and thereby induce a specific antitumor immune response and overcome immunosuppression, as well as improving the immune response. The targeted delivery of antigens and adjuvants to DCs *in vivo* has been an important method for developing DC vaccines. Compared to traditional exogenous adjuvants such as Freund’s adjuvant, aluminum salts, or emulsion-based adjuvants, endogenous adjuvants have significant advantages, such as higher activity, higher safety, longer-lasting effects, better preservation and injection, and lower damage. According to Cheng et al. (110), sEVs generated by M1-polarized macrophages can be used as vaccine adjuvants due to their capacity to connect lymph nodes and promote the expression of inflammatory T helper cell type 1 cytokines, which results in local inflammation and sustained release of antigen at injection sites.

A variety of cancer vaccines have been studied and entered clinical use in a variety of diseases, such as sipuleucel-T (Provenge, Dendreon) has been applied to treat asymptomatic metastatic debulking resistant prostate cancer (mCRPC) and is the only DC vaccine approved by the FDA (111). Cancer vaccines in non-small cell lung cancer (112–114) and melanoma (115, 116) have been studied in phase I and II clinical trials with excellent results. Other well-studied examples of cancer vaccine applications are glioblastoma (117, 118), breast cancer (119), ovarian cancer (120), etc. However, studies in hepatocellular carcinoma are still relatively few. In a study by Lu et al. (121), the authors loaded AFP onto DCs-derived exosomes, resulting in the induction of a strong specific immune response and a remodeling of the tumor microenvironment in HCC mice, which led to significant retardation of tumor growth and prolonged survival.

The manufacturing of cancer vaccines still faces numerous challenges, including how to elicit a strong and durable immune response due to the weak immunogenicity of autologous tumor antigens and the highly immunosuppressed microenvironment. According to Rao et al. (122), compared to cell lysates (DClys) pulsed on DCs, exosomes from HCC cells targeting pulsed DCs (DCTEX) induced significantly stronger immune responses. They reshaped the tumor immune microenvironment, resulting in HCC-specific cytolysis and tumor growth inhibition. However, the mechanism is unclear, and only delayed tumor growth was observed in the experiments, with no complete tumor regression, so further studies are needed. In the study by Zuo et al. (123), a high mobility group nucleosome-binding protein 1 (HMGN1) functional domain loaded on the exosome anchoring peptide triggered durable antitumor immunity and tumor suppression. Individualized treatment of cancer vaccines is also a huge challenge. Zuo et al. (124) Combining DC-derived exosomes (DEX) with HCC targeting peptide (P47-P), alpha-fetoprotein epitope (AFP212-A2), and high mobility group nucleosome-binding protein 1 (N1ND-N) domains combined and loaded together as immune adjuvants for DC recruitment and activation. The method enables personalized immunotherapy of tumors without identifying tumor antigens, which is a promising treatment technique.

## Discussion

Exosomes are tiny vesicles that can be produced by normal, pathological, or cancerous cells. Exosomes play a key role in substance delivery and signaling and are essential to maintain the stability of the body. During tumor development, intercellular delivery of proteins, lipids, and nucleic acids from exosomes can alter the TME, promote angiogenesis, modulate drug resistance, and increases HCC cell proliferation, invasion, and metastasis of HCC. Simultaneously, exosomes regulate several physiological and pathological processes of the body and can be used as diagnostic biomarkers of disease and vehicles for drug therapy. Exosomes have great potential, but many issues remain unresolved and require further research despite their great potential. A number of scientific questions remain unanswered regarding exosome identification, isolation, purification, storage, and characterization, and their technology has not yet been standardized, so further improvement and optimization are required for better clinical applications. Moreover, although current studies have demonstrated the great potential of exosomes in HCC diagnosis and treatment, further explanation of the molecular mechanisms affecting the progression of HCC and the role of exosomes in it is needed in the future to use exosomes as a more efficient diagnostic tool.

## Author contributions

GW, GL and MZ wrote the paper. HM conceived the idea and supervised the manuscript. GW and GL contributed equally to this work. All authors contributed to the article and approved the submitted version.

## Funding

The study was partly supported by the National Natural Science Foundation of China (82070637).

## Acknowledgments

We thank Editeg (https://www.editeg.com// ) for editing the English text of a draft of this manuscript.

## Conflict of interest

The authors declare that the research was conducted in the absence of any commercial or financial relationships that could be construed as a potential conflict of interest.

## Publisher’s note

All claims expressed in this article are solely those of the authors and do not necessarily represent those of their affiliated organizations, or those of the publisher, the editors and the reviewers. Any product that may be evaluated in this article, or claim that may be made by its manufacturer, is not guaranteed or endorsed by the publisher.
